# Is Immune Therapy Plus Chemotherapy More Effective Than Immune Therapy Alone for Unresectable Recurrent Nasopharyngeal Carcinoma?

**DOI:** 10.3389/fimmu.2021.762663

**Published:** 2021-10-29

**Authors:** Xin Zhou, XiaoShuang Niu, PeiYao Liu, Dan Ou, Yi Zhu, XiaoShen Wang

**Affiliations:** ^1^ Department of Radiation Oncology, Fudan University Shanghai Cancer Center, Shanghai, China; ^2^ Department of Oncology, Shanghai Medical College, Fudan University, Shanghai, China; ^3^ Shanghai Key Laboratory of Radiation Oncology, Shanghai, China; ^4^ Department of Radiation Oncology, Eye and ENT Hospital, Fudan University, Shanghai, China

**Keywords:** immune checkpoint inhibitor, recurrent nasopharyngeal carcinoma, chemotherapy, unresectable, progression-free survival, objective response rate

## Abstract

**Objective:**

To evaluate whether the combination of immune checkpoint inhibitor (ICI) with chemotherapy is more effective than ICI alone in the treatment of recurrent, locoregionally advanced, unresectable nasopharyngeal carcinoma (RAU-NPC), which has progressed after second line chemotherapy.

**Methods and materials:**

Patients with RAU-NPC that progressed after second chemotherapy were prescribed ICI once every 3 weeks, either alone or combined with chemotherapy at the discretion of treating physicians, until confirmed disease progression, unacceptable toxicity, or voluntary withdrawal. The primary endpoint was the objective response rate (ORR). The secondary endpoints included safety, duration of response (DOR), and progression-free survival (PFS).

**Results:**

From June 2016 to July 2021, 28 patients were enrolled in this study.21 patients received ICI plus chemotherapy, and 7 patients received ICI alone. Altogether, there were 7 (25%) complete response (CR) and 12 (42.8%) partial response (PR), respectively. Stable disease (SD) and progressive disease (PD) were defined in 4 (14.3%) and 5 (17.8%) cases, respectively. The ORR was 19 out of 28 (67.8%). The disease control rate (DCR) was 23 out of 28 (82.1%).Two patients (28.6%) in the ICI alone group and five (23.8%) in the combination group achieved CR (P=0.801). 2 patient (28.6%) in the ICI alone group and 10 (47.6%) in the combination group achieved PR (P=0.378). With a median follow-up of 16 months (2-61 months), five patients terminated ICI due to disease progression, one patient was lost to follow-up, and the remaining 22 patients continued with ICI. Neither the median PFS nor the median DOR was reached. All observed adverse events were defined as ≤ Grade 2.

**Conclusion:**

ICI alone or combined with chemotherapy demonstrated promising antitumor activity in RAU-NPC patients that progressed after second line chemotherapy, with a low toxicity profile. Compared with ICI alone, chemotherapy plus ICI did not improve CR or PR in our study.

## Introduction

The widespread application of intensity-modulated radiation therapy (IMRT) and multidisciplinary treatment (MDT) strategies for nasopharyngeal carcinoma (NPC), excellent locoregional control rates have been achieved in the past decade, although 10%–20% of patients continue to suffer from local or nodal recurrence after primary treatment (1, 2). For resectable locally recurrent NPC, treatment modalities such as endoscopic surgery and re-irradiation with IMRT are recommended by the National Comprehensive Cancer Network guidelines. A multicenter, randomized, controlled, phase 3 study by Liu et al. demonstrated that endoscopic nasopharyngectomy led to a higher 3-year overall survival (OS) rate(85.8% vs. 68.0%), better quality of life (QoL), and a lower rate of treatment-related lethal complications (5% vs. 20%) compared with salvage IMRT (3), suggesting that nasopharyngectomy is superior to re-irradiation in resectable disease.

In practice, only a small proportion of recurrent NPC is suitable for endoscopic nasopharyngectomy, and a large majority of cases are defined as unresectable on diagnosis. The treatment for recurrent, locoregionally advanced, and unresectable nasopharyngeal carcinoma (RAU-NPC) remains challenging. Salvage IMRT is the most commonly preferred treatment modality for RAU-NPC. The reported five-year survival rates ranged from 28%–60%, depending on the disease stage. However, radiotherapy-related lethal toxicities should not be ignored. According to a recent meta-analysis (4), Grade 5 toxicities were observed in 33% of patients, with mucosal ulcer/necrosis and massive hemorrhage reported as the most common severe toxicities, followed by dysphagia and cerebral radiation necrosis (4, 5), resulting in long-term deterioration in patients’ QoL.

Due to the high incidence of Grade 5 adverse events caused by a second radiotherapy (RT) course, alternative treatment with chemotherapy is increasingly recommended by physicians. A retrospective study demonstrated that salvage RT and chemotherapy led to similar 2-year OS rates (55%) (6). Liu et al. performed a case–control study to evaluate the outcomes among cases of RAU-NPC treated with or without re-irradiation (7). They found that patients receiving salvage RT experienced better local tumor control than those treated with chemotherapy alone (49.8% vs. 39.0%). However, the two-treatment modality yielded similar 5-year OS rates (27.5% vs. 23.4%). The absence of survival benefits associated with a second RT course was due to radiation-related complications, which led to 13 deaths attributed to radiation-related injuries. Therefore, treatment toxicity is a crucial consideration when decided on a salvage treatment strategy for RAU-NPC.

In recent years, with a deeper understanding of tumor immunology, programmed cell death protein-1 (PD-1) blockade immunotherapy has emerged as a promising treatment modality, alongside surgery, radiotherapy, and chemotherapy (8). Among recurrent or metastatic NPC (RM-NPC) patients with poor prognosis who have progressed on second-line chemotherapy and beyond, a series of prospective phase II studies have examined the efficacy of anti-PD-1 immune checkpoint inhibitors (ICIs) alone and demonstrated an objective response rate (ORR) ranging from 20.5% to 34.1% (9–12). The combination of camrelizumab plus gemcitabine and cisplatin as a first-line treatment in RM-NPC yielded an ORR of up to 91% and a median progression-free survival (PFS) rate of 10.2 months (12).To date, however, no study has examined ICI therapy, with or without chemotherapy, in RAU-NPC. Therefore, we performed this study to analyze the safety and efficacy of ICI alone or in combination with chemotherapy for RAU-NPC in a real-world setting.

## Materials and Methods

### Patients

Patients were consecutively enrolled in real-world clinical practice. The inclusion criteria were: (1) pathologically confirmed recurrent NPC, including World Health Organization (WHO) type I/II/III pathology, or radiologically diagnosed recurrent NPC; (2) multidisciplinary team (MDT)-defined locoregionally advanced and unresectable disease;(3) age 18–80 years with Eastern Cooperative Oncology Group (ECOG) performance status of 0 or 1;(4) measurable baseline disease on magnetic resonance imaging (MRI) according to Response Evaluation Criteria in Solid Tumors (RECIST version 1.1); (5) disease progression after at least one prior line of platinum-based chemotherapy; 6) adequate organ function as determined by laboratory tests; and (7) receiving at least two cycles of ICI.

The exclusion criteria were: (1) recurrent NPC with another progressive malignancy or that required active treatment; (2) previous treatment with any other ICI; (3) active autoimmune disease; (4) interstitial lung disease; and (5) corticosteroid therapy within one week of study start.

### Chemotherapy and Immunotherapy

Previous publications have shown that a series of cytotoxic agents demonstrated good antitumor effect against NPC. The most commonly adopted regimens included cisplatin/5-fluorouracil (5-FU; PF), docetaxel/cisplatin (TP), docetaxel/cisplatin/5-FU (TPF), and gemcitabine/cisplatin (GP) (13). For patients with recurrent and metastatic NPC who progressed after platinum-based chemotherapy, capecitabine or S-1 alone has been shown to be an effective salvage regimen (14, 15). Available ICIs included seven anti-PD1 checkpoint inhibitors (pembrolizumab, nivolumab, camrelizumab, toripalimab, sintilimab, tislelizumab, and penpulimab), all have been shown with confirmed efficacy. No standard chemotherapy regimen or fixed ICI was utilized in the present study. Salvage chemotherapy was chosen individually, based on each patient’s age, performance status, and previous chemotherapy. For example, in case of disease progression following a PF or TP regimen, salvage chemotherapy could be GP or gemcitabine alone. For those with previous GP and TP regimen, capecitabine or S-1 alone might be considered. ICI was prescribed at the discretion of the treating physicians and based on the availability. ICI was administered every three weeks *via* intravenous infusion until intolerable toxicity, disease progression, or voluntary withdrawal. Altogether, 7 patients received ICI alone, and 21 patients received ICI combined with chemotherapy. Detailed treatment information was shown in **Table 1**.

**Table 1:** Detailed treatment and response information for the current study.

Treatment modality	No. of patients	CR	PR	SD	PD	HPD
GP plus ICI	5	1	2	0	1	1
TP plus ICI	2	0	1	1	0	0
Gemcitabine and S-1 plus ICI	5	2	2	1	0	0
Gemcitabine plus ICI	2	0	2	0	0	0
S-1 plus ICI	5	1	2	1	1	0
Capecitabine plus ICI	2	1	1	0	0	0
ICI alone	7	2	2	1	1	1

GP, gemcitabine/cisplatin; ICI, immune checkpoint inhibitor; TP, docetaxel/cisplatin; CR, complete response; PR, partial response; SD, stable disease; PD, progressive disease; HPD, hyperprogressive disease.

### Outcome and Safety Assessments

Prior to treatment, all patients received a comprehensive physical examination (PE), a thorough laboratory test, and an imaging evaluation. To record the safety profile and antitumor effects of ICI with or without chemotherapy, a PE and laboratory test was required before each dose, and an imaging evaluation was performed every two cycles throughout the treatment course.

Tumor response was assessed according to RECIST v1.1 and immune-related RECIST, which was performed by an independent radiologic review committee (IRC) and the study investigators. Adverse events were graded according to National Cancer Institute Common Terminology Criteria (CTCAE) version 4.0.

The primary endpoint was ORR. The secondary endpoints included safety, duration of response (DOR), disease control rate (DCR), and PFS.

### Statistical Analysis

All statistical analyses were performed using IBM SPSS Statistics for Windows, version 25.0 (IBM Corp., Armonk, NY, USA). PFS was defined as the date of treatment initiation to the date of the first failure at any site, death by any cause, or patient censoring at the date of the last follow-up. PFS and DOR were calculated using the Kaplan-Meier method.

## Results

### Patient Population

From July 2016 to July 2021, a total of 28 patients with RAU-NPC were enrolled in this study. The male-to-female ratio was 3:1. Seven cases received ICI alone, and 21 cases received ICI plus chemotherapy. Two patients had failure disease after the second course of radiotherapy. Detailed characteristics of the 28 patients are listed in **Table 2**.

**Table 2:** Clinical characteristics of the 28 patients.

Variable	Results
Sex	
Male	21
Female	7
Age	median 51 (18–80)
ECOG PS	
0	18
1	10
Recurrent sites	
Nasopharynx	17
Neck	5
Both	6
Prior lines of chemotherapy	
2	23
≥3	5
Prior radiotherapy	
1 course	26
2 courses	2
Salvage treatment	
ICI alone	7
ICI with chemotherapy	21
No. of ICI cycles	19(2-89)

ECOG PS, Eastern Cooperative Oncology Group performance status; ICI, immune checkpoint inhibitor

### Antitumor Activity

**Figure 1:**
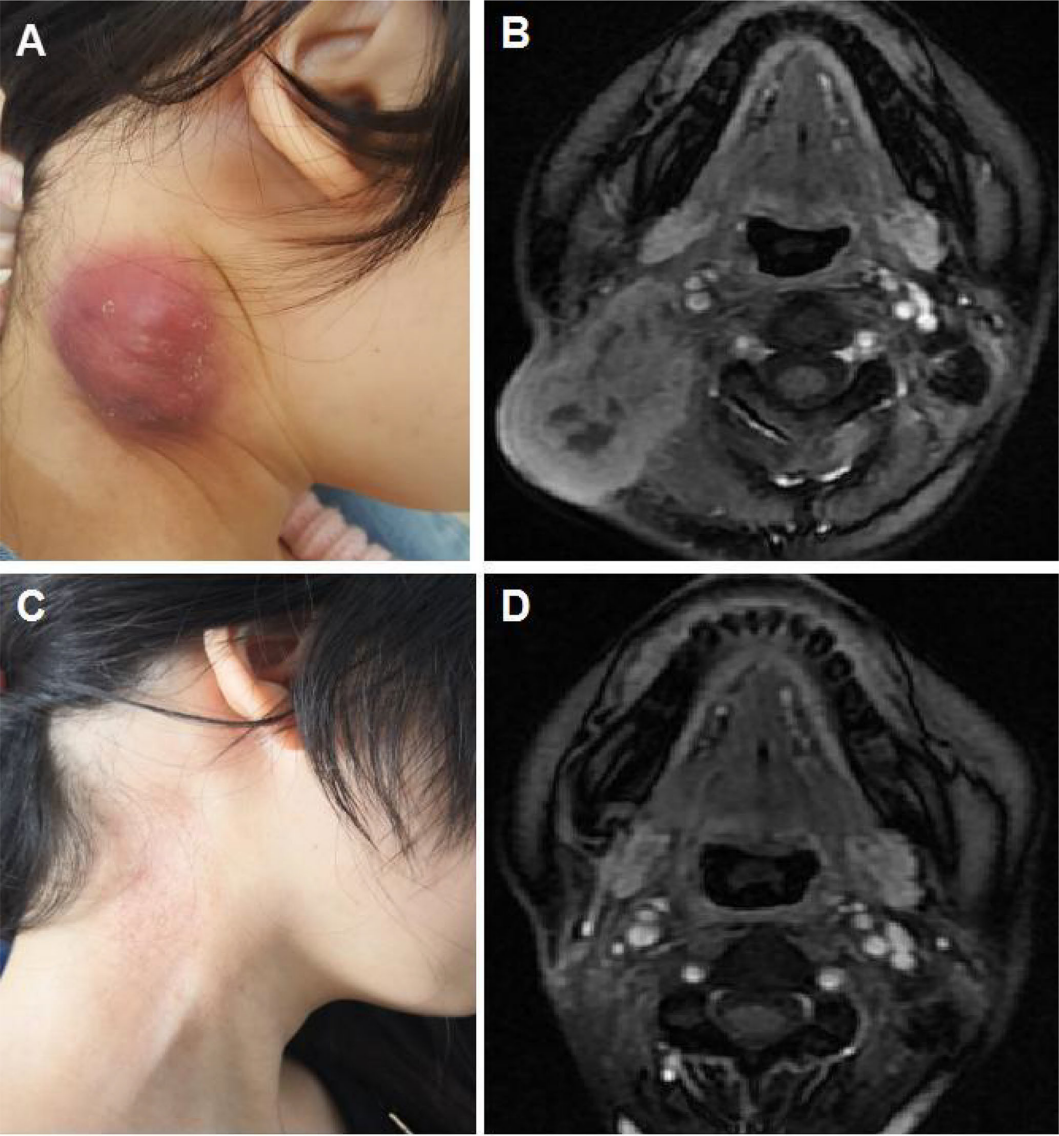
Illustration of a typical recurrent nasopharyngeal carcinoma (NPC) case showing complete response after immune checkpoint inhibitor (ICI) treatment alone. **(A)** Baseline appearance of an 18-year-old girl with recurrent NPC who progressed after fifth-line chemotherapy. **(B)** Baseline magnetic resonance imaging (MRI) demonstrating a large mass in the right neck. **(C)** Appearance of the 18-year-old girl after 4 cycles of ICI alone showing complete tumor response. **(D)** MRI illustrating the complete response of the mass in the right neck after 4 cycles of ICI alone.

**Figure 2:**
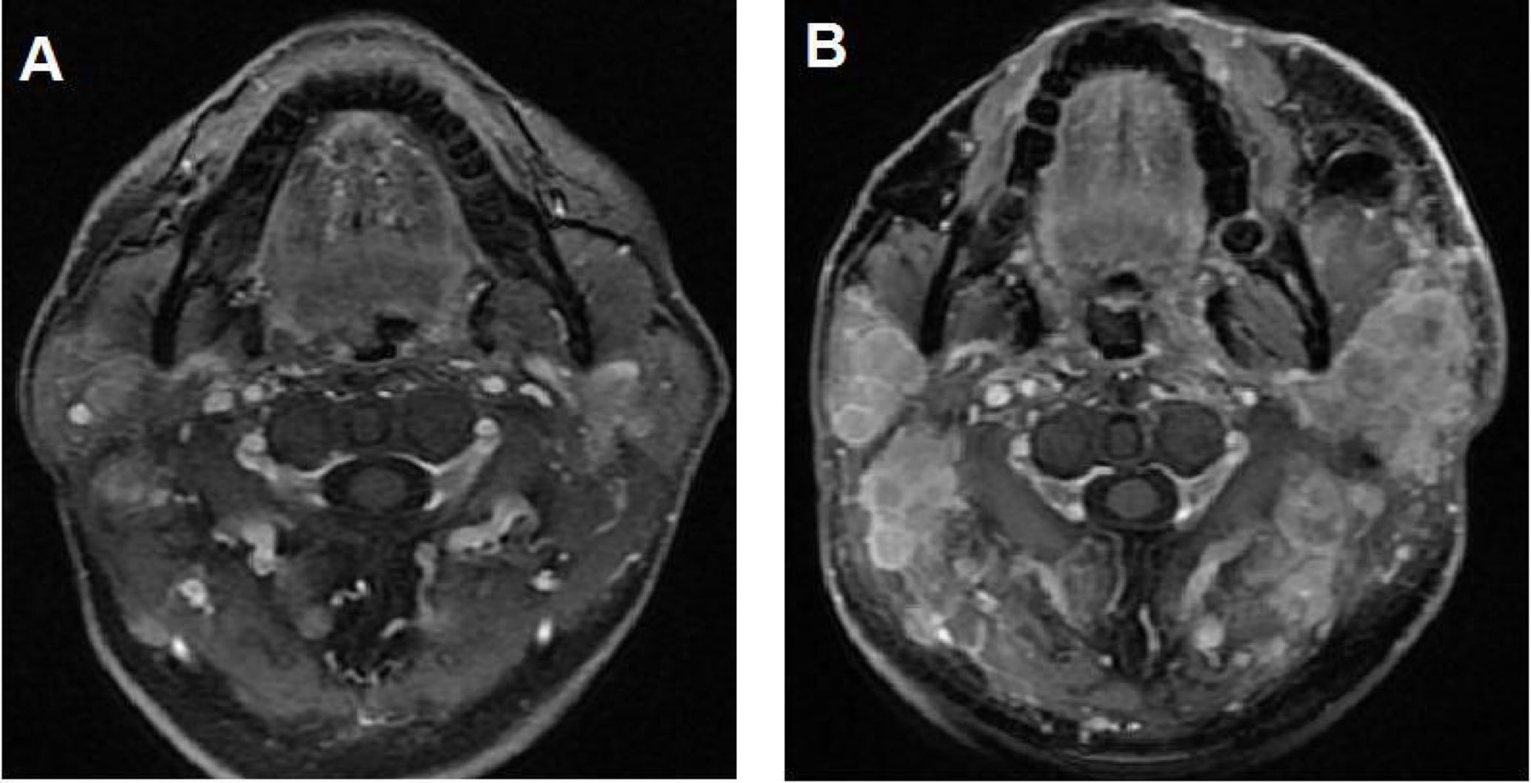
Illustration of a typical recurrent nasopharyngeal carcinoma (NPC) case showing hyperprogressive disease (HPD) after immune checkpoint inhibitor (ICI) treatment alone. **(A)** Baseline magnetic resonance imaging (MRI) demonstrating multiple metastatic disease lesions in the bilateral neck. **(B)** MRI showing HPD in the bilateral neck after 2 cycles of ICI alone.

Altogether, 7 patients (25%) achieved complete response (CR), including two (28.6%) in the ICI alone group and five (23.8%) in the combination treatment group (P=0.801). Twelve patients (42.8%) were defined as partial response (PR), with 2 (28.6%) in the ICI alone group and 10 (47.6%) in the combination group (P=0.378). The ORR was 19 out of 28 (67.8%). Stable disease (SD) and progressive disease (PD) were identified in 4 (14.3%) and 5 (17.8%) patients, respectively. DCR was 23 out of 28 cases (82.1%). Among the 5 cases with PD, 2 were defined as hyperprogressive disease (HPD), with 1 in the ICI alone group and another in the combination group. A detailed distribution of the tumor responses is shown in **Table 1**. Compared with ICI alone, chemotherapy plus ICI did not improve CR or PR in our study. Typical MRI images of CR and HPD are illustrated in **Figures 1**, **2**, respectively.

Up to July 2021, the median follow-up time from the start date of immunotherapy for all patients was 16 months (2-61 months). Five patients had terminated ICI due to disease progression, one patient was lost to follow-up, and the remaining 22 patients continued to receive ICI. Neither median PFS nor median DOR was reached in the study.

### Safety

All patients completed at least two cycles of ICI. Due to the use of different chemotherapy regimens and ICI from different pharmaceutical companies, a series of sporadic adverse events were observed in our study. But there were no ≥ Grade 3 adverse events among the 28 patients. All toxicities were defined as ≤ Grade 2. The details regarding adverse effects are listed in **Table 3**.

**Table 3:** Adverse events in the study.

Variable	ICI alone	ICI + chemotherapy
Hypothyroidism	3	8
Anemia	0	5
Liver dysfunction	1	5
Kidney dysfunction	0	3
Asthenia	1	6
Leukopenia	0	4
Thrombocytopenia	0	6
Rash	2	5
Diarrhea	0	2
Anorexia	0	9
Hand and foot syndrome	0	4
RCCEP	1	2
Pruritus	1	3

RCCEP, reactive cutaneous capillary endothelial proliferation; ICI, immune checkpoint inhibitor.

## Discussion

Up to date, no optimal treatment modality for recurrent NPC has been established. Salvage strategies include endoscopic surgery, re-irradiation with IMRT or intensity-modulated proton therapy (IMPT), chemotherapy plus anti-epidermal growth factor receptor (EGFR) molecular-targeted therapy, or chemotherapy combined with immunotherapy, depending on the recurrent disease stage. Salvage endoscopic surgery has been applied for early-stage recurrent NPC, with promising local control (3). However, for the majority of locally recurrent NPC with extensive lesions, the efficacy of surgical resection remains limited, and salvage IMRT or IMPT remains a widely adopted management. Although the 5-year local control rate has been reported as high as 61%, it failed to translate into a better OS because fatal complications negated the benefits of improved tumor control. The 5-year OS rate was only 27.5% due to a high incidence of lethal Grade 5 toxicities (5). According to a recent meta-analysis examining re-irradiation for recurrent NPC (4), Grade 5 toxicities were observed in one-third of patients, with the most common severe effects being mucosal necrosis and massive hemorrhage, followed by dysphagia, aspiration pneumonia, and radiation encephalopathy. Platinum-based doublet chemotherapy, such as TP or GP, represents another widely applied modality for advanced, recurrent disease (16, 17). The GP regimen has been recommended as the standard first-line treatment for recurrent/metastatic (R/M) NPC based on a multicenter, randomized, phase-3 clinical trial (18). However, the median PFS was only 7 months.

Due to the limited improvements following chemotherapy, an urgent need exists for the development of new treatment regimens to treat R/M-NPC that can potentially improve PFS and OS. For R/M-NPC that progressed after standard platinum-based chemotherapy, ICI alone showed the ORR ranging from 20.5% to 34.1%, and the PFS ranging from 2.8 months to 10.7 months in different studies (9–12). However, no ongoing studies have focused on the actual role of ICI in RAU-NPC. To our knowledge, this is the first study to explore the safety and antitumor efficacy of ICI alone or in combination with chemotherapy in RAU-NPC that progressed after second or subsequent-line chemotherapy. Our study suggested that ICI with or without chemotherapy has promising antitumor effects (ORR: 67.8%,and DCR:82.1%) with tolerable toxicities (no ≥ Grade 3 adverse events). The DOR to ICI in our study was very long, with a median follow-up of 16 months, neither median PFS nor median DOR was reached. Similar evidence was reported in a prospective study demonstrating that ICI significantly improved 6-month PFS and OS for R/M-NPC patients with progressed disease after first or subsequent-line therapy, and another study showed that the combination of camrelizumab with GP as first-line treatment in R/M-NPC led to a 91% ORR and a median PFS of 10.2 months (12). However, neither the CR nor PR rate reached significance in our study. The CR rates in the ICI alone group and combination group were 28.6% and 23.8%, respectively (P=0.801). The PR rates in the ICI alone group and combination group were 28.6% and 47.6%, respectively (P=0.378).

The present study has some limitations. First, this study was a real-world study, neither the chemotherapy regimen nor the ICI was consistent among patients. In addition, some patients received ICI alone while others received ICI plus chemotherapy. Due to the limited sample size, reliable comparisons between the two groups were difficult. Second, the status of PD-L1 expression on tumor cells or immune cells was unavailable at the time of this study, therefore, the impact of PD-L1 expression on the efficacy of immunotherapy was challenging to evaluate. Third, our study included previously heavily treated patients with a maximum of five lines of salvage chemotherapy, whether chemotherapy plus ICI as the first-line therapy would lead to a better outcome warrants further exploration. At present, we are conducting a prospective study to investigate the feasibility of postponing or omitting re-irradiation (RE) after tislelizumab (TI) plus chemotherapy (C) for unresectable (U), recurrent locoregionally advanced (LA) NPC (RETICULA-NPC, NCT 04921995).

In conclusion, ICI alone or combined with chemotherapy demonstrated promising antitumor activity in RAU-NPC patients who had progressed after at least second line chemotherapy in a real-world setting, with a low toxicity profile. Compared with ICI alone, chemotherapy plus ICI did not improve CR or PR in our study. Well-designed prospective, randomized, controlled phase 3 trials should be conducted for further verification.

## Data Availability Statement

The original contributions presented in the study are included in the article/supplementary material. Further inquiries can be directed to the corresponding author.

## Ethics Statement

The studies involving human participants were reviewed and approved by the ethics committee of Fudan University, EENT Hospital. The patients/participants provided their written informed consent to participate in this study.

## Author Contributions

XZ, XN, PL, and DO contributed equally to this work. They were in charge of selecting the proper patients and carrying out the treatment, follow-up, and fill-out of the case report form. YZ performed the statistical work. XW offered the proposal and wrote the manuscript. All authors contributed to the article and approved the submitted version.

## Funding

This study was supported by the Medical Guidance Project of the Shanghai Science and Technology Commission [number 19401931700 and number 21Y11900300].

## Conflict of Interest

The authors declare that the research was conducted in the absence of any commercial or financial relationships that could be construed as a potential conflict of interest.

## Publisher’s Note

All claims expressed in this article are solely those of the authors and do not necessarily represent those of their affiliated organizations, or those of the publisher, the editors and the reviewers. Any product that may be evaluated in this article, or claim that may be made by its manufacturer, is not guaranteed or endorsed by the publisher.
